# Watching Movies Unfold, a Frame-by-Frame Analysis of the Associated Neural Dynamics

**DOI:** 10.1523/ENEURO.0099-21.2021

**Published:** 2021-07-09

**Authors:** Anna M. Monk, Daniel N. Barry, Vladimir Litvak, Gareth R. Barnes, Eleanor A. Maguire

**Affiliations:** Wellcome Centre for Human Neuroimaging, University College London Queen Square Institute of Neurology, University College London, London WC1N 3AR, United Kingdom

**Keywords:** ERFs, hippocampus, MEG, movie events, scenes, sequences

## Abstract

Our lives unfold as sequences of events. We experience these events as seamless, although they are composed of individual images captured in between the interruptions imposed by eye blinks and saccades. Events typically involve visual imagery from the real world (scenes), and the hippocampus is frequently engaged in this context. It is unclear, however, whether the hippocampus would be similarly responsive to unfolding events that involve abstract imagery. Addressing this issue could provide insights into the nature of its contribution to event processing, with relevance for theories of hippocampal function. Consequently, during magnetoencephalography (MEG), we had female and male humans watch highly matched unfolding movie events composed of either scene image frames that reflected the real world, or frames depicting abstract patterns. We examined the evoked neuronal responses to each image frame along the time course of the movie events. Only one difference between the two conditions was evident, and that was during the viewing of the first image frame of events, detectable across frontotemporal sensors. Further probing of this difference using source reconstruction revealed greater engagement of a set of brain regions across parietal, frontal, premotor, and cerebellar cortices, with the largest change in broadband (1–30 Hz) power in the hippocampus during scene-based movie events. Hippocampal engagement during the first image frame of scene-based events could reflect its role in registering a recognizable context perhaps based on templates or schemas. The hippocampus, therefore, may help to set the scene for events very early on.

## Significance Statement

Our experience of the world is much like watching a movie. Although it appears to be seamless, it is in fact composed of individual image frames that we perceive between eye blinks. The hippocampus is known to support event processing, but questions remain about whether it is preferentially involved only when events are composed of scenes that reflect the real world. We found that a set of brain regions including the hippocampus was engaged during the first image frame of scene-based events compared with highly matched events composed of abstract patterns. This suggests that the hippocampus may set the scene for an event very early on.

## Introduction

We generally perceive the world as a series of visual snapshots punctuated by eye blinks and saccades. With parallels in terms of how the individual frames of a movie appear to be continuous (Tan, 2018), somehow these separate images become linked together, such that we have a sense of the seamless unfolding of life and events (Cutting, 2005; Magliano and Zacks, 2011). These dynamic events are central to our lived experience, be that during “online” perception, or when we recall the past or imagine the future.

Here, we defined an event as a dynamic, unfolding sequence of actions that could be described in a story-like narrative. Functional MRI (fMRI) has helped delineate the brain areas involved in supporting event processing (Zacks et al., 2001; Hasson et al., 2008; Lehn et al., 2009; Summerfield et al., 2010; Reagh et al., 2020), a salient example being the events captured in autobiographical memories. When people recollect these past experiences, a distributed set of brain areas is engaged, including the hippocampus, parahippocampal, retrosplenial, parietal, and ventromedial prefrontal cortices (Maguire, 2001; Svoboda et al., 2006; Cabeza and St Jacques, 2007; Spreng et al., 2009). Interestingly, two key elements of events, individual scene snapshots (Hassabis et al., 2007a; Zeidman et al., 2015) and sequences (Kumaran and Maguire, 2006; Lehn et al., 2009; Schapiro et al., 2016; Liu et al., 2019), engage several of the same brain regions, including the hippocampus. Aligning with these fMRI findings, impairments in recalling past events (Scoville and Milner, 1957; Rosenbaum et al., 2005; Kurczek et al., 2015), imagining single scene images (Hassabis et al., 2007b), and processing sequences (Mayes et al., 2001; Dede et al., 2016) have been documented in patients with bilateral hippocampal damage.

While much has been learned about event processing from fMRI and neuropsychological studies, we still lack knowledge about the precise temporal dynamics associated with unfolding events. This is not surprising given the temporal lag of the fMRI BOLD signal. By contrast, the finer temporal resolution of evoked responses measured by magnetoencephalography (MEG) and electroencephalography (EEG) offers a means to establish the millisecond-by-millisecond neural dynamics associated with events as they evolve. There are relatively few MEG/EEG studies of event processing. Investigations have typically used viewing of movies or television shows as event stimuli to examine the consistency of neural activity patterns across participants (Lankinen et al., 2014; Chang et al., 2015; Betti et al., 2018; Chen and Farivar, 2020), or to assess segmentation of such stimuli into discrete events (Silva et al., 2019). However, the fundamental underlying temporal dynamics of event processing remain essentially unaddressed.

Extended events, as represented in movies or autobiographical memories, involve visual imagery from the real world and, as noted, the hippocampus is frequently engaged in this context. It is unclear, however, whether it would be similarly responsive to unfolding events that involve abstract imagery. One theoretical position suggests that the hippocampus may be especially attuned to scenes (Maguire and Mullally, 2013), which we define simply as individual visual images reflecting the real world. Consequently, here, we compared the watching of closely-matched scene-based movie events and non-scene movie events during MEG, with a particular interest in the hippocampal response.

To do this, we created a set of short, simple cartoon-like movies each of which depicted an event. Each event was a self-contained vignette, and was composed of a series of individual images. These individual images were linked such that each image led on to the next, thereby depicting an activity that unfolded over 22.4 s. In essence, the events were digital versions of a flip-book, composed of individual images which, when presented as a sequence, showed the progression of an activity. We devised two types of movie events. In one, each image frame within a movie was a simple scene reflecting the real world, while in the other condition each image frame comprised abstract shapes. The two event types were visually very similar, and both involved unfolding sequences of individual image frames that could be described in a story-like narrative. By leveraging the high temporal resolution of MEG, we could examine each image, allowing us to better understand how a sequence of separate images evolves neurally to give rise to the experience of a seamless event. Moreover, by comparing the two event types, we could address the question of whether or not the hippocampus was preferentially engaged by scene-based events, with relevance for theories of hippocampal function.

## Materials and Methods

### Participants

Twenty-one healthy human participants (11 females; mean age 23.42 years, SD 4.51) took part in the study. All participants had normal or corrected vision, and provided written informed consent to participate in the study (protocol #1825/005) as approved by the local Research Ethics Committee.

### Stimuli

Short visual movies of events were created by hand using the animation program Stykz 1.0.2 (https://www.stykz.net), each composed of 16 individually drawn image frames presented in a sequence. Each of these 16-image movies lasted 22.4 s. Each event was self-contained and was not part of a larger story. An image comprised a combination of straight lines and circles that created simple line imagery that was easily interpretable. Images were all grayscale to keep the luminance contrast low, and this was invariant across frames and between conditions. A pixelated gray background for all frames was created in the image manipulation software program GIMP 2.8 (https://www.gimp.org).

**Figure 1.**
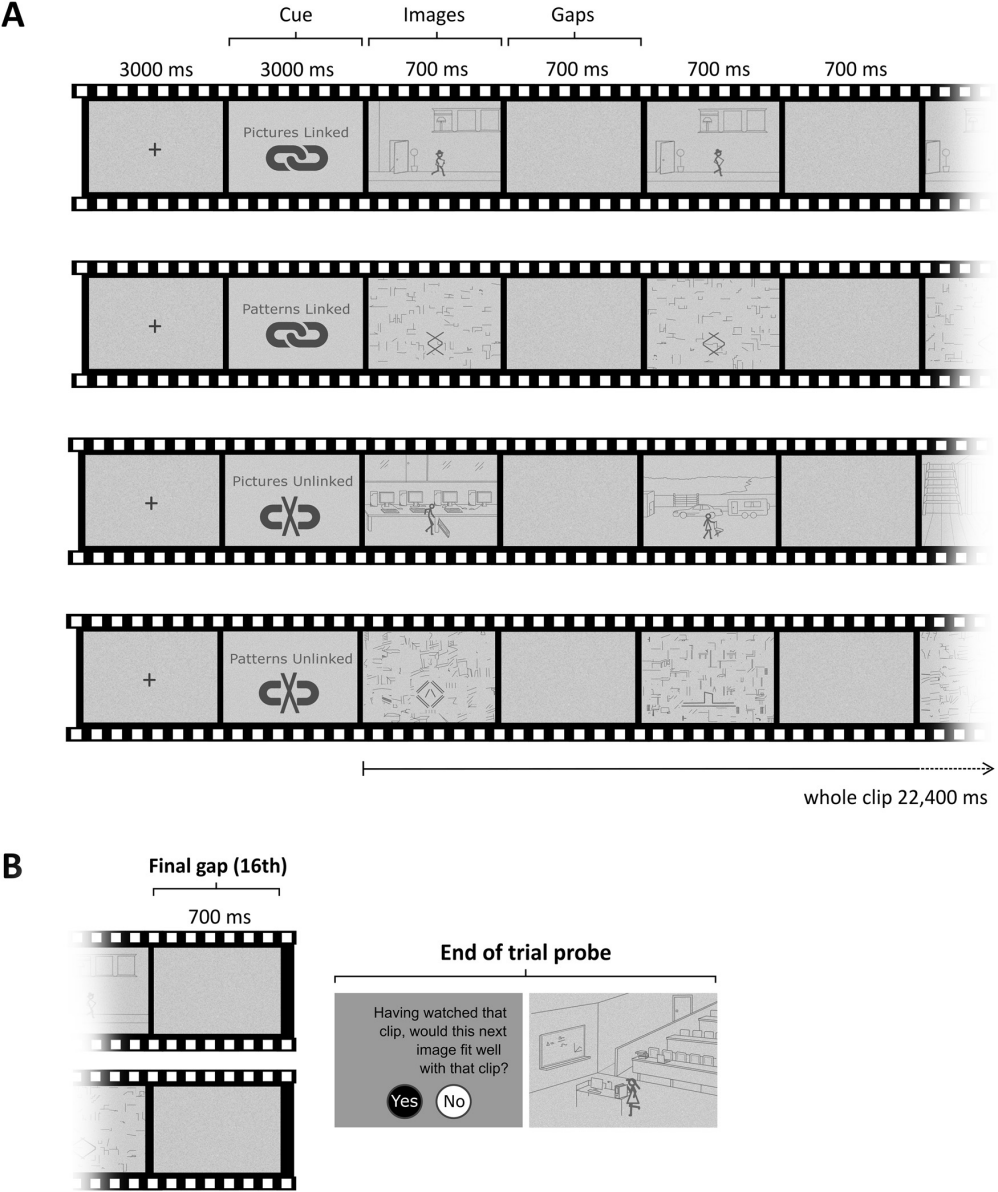
Experimental paradigm. ***A***, Schematic of the trial structure common to all movies, with examples of each condition. Each image frame was followed by a gap frame. ***B***, A probe question occasionally followed the completion of a clip to assess participants’ engagement with the task.

There were two main stimulus types (Fig. 1*A*, upper two panels). In one case, each image frame within a movie event was a simple scene (pictures), while in the other, each image frame was composed of abstract shapes (patterns). In both cases, incremental changes in the stimuli image-to-image showed a progression of activity that connected every image frame over the course of a 22.4-s clip, resulting in two event conditions called pictures-linked and patterns-linked. Pictures-linked movies contained temporally related scenes unfolding over time such that a scene-based event was perceived. A stick-figure character in the center of the image performed a series of brief activities involving a single object that appeared in every frame of the movie. Each movie clip portrayed a different stick-figure paired with a different object with which they interacted. The environment for each movie was a simple representation of an indoor (50% of clips) or outdoor (50% of clips) scene. Other background objects were included to give a sense of perspective and movement. Patterns-linked movies contained temporally unfolding patterns that matched the evolving nature of pictures-linked movies, where each showed a novel abstract shape that underwent numerous simple mechanical changes, such as a rotation in a particular direction. The result was that a non-scene event was perceived. See https://vimeo.com/megmovieclips for dynamic examples of the movies.

The activity depicted in an event could be subdivided into steps. For example, for the event where the overall activity was skate-boarding (see https://vimeo.com/megmovieclips), the individual steps included: (1) the stick-figure skate-boards over a ramp; (2) lands on the ground; (3) skate-boards on the ground past some shopping carts; and (4) steps off the skate-board and picks it up. Each of these steps, or “subevents,” were ≥1 s in duration. Each of the patterns-linked events also contained subevents. For the example provided here: https://vimeo.com/megmovieclips, (1) three diamond shapes are nested within each other; (2) the three diamond shapes start to separate out; (3) the three diamonds become separate and the lines comprising the diamonds become thicker; and (4) the diamond shapes all rotate to the right.

There was one-to-one matching between the stimuli of the two conditions. For each image frame in a patterns-linked movie, the number of pixels composing the central shape was matched with the number of pixels composing the stick-figure and its paired object from a corresponding pictures-linked movie. Pictures-linked background images (minus the stick-figure and object) were scrambled to form the individual backgrounds of patterns-linked image frames. The number of frames it took for a particular pattern’s movement to unfold (e.g., completion of one rotation of a shape) corresponded to the same number of frames it took for a stick-figure to accomplish a subactivity (e.g., the stick-figure skate-boarded over a ramp), so that the pace of subevents was matched between conditions. An average of four subevents occurred per linked movie. There were 10 unique movies for each stimulus type.

There were two control conditions (Fig. 1*A*, lower two panels), each with 10 movie clips. Each movie was composed of a series of unique and separate unrelated image frames such that no evolving event could be conceived. Pictures-unlinked movies contained separate scenes for each image frame, and in total there were 160 unique scenes, twenty different stick-figures and 152 unique objects. For example, in one pictures-unlinked movie (see web link), the first image shows a stick-figure in an Internet café, the next image shows a different stick-figure in a trailer park, and each of the remaining images are similarly unrelated to one another. Patterns-unlinked movies were composed of a series of unrelated abstract shapes. In total, there were 160 unique shapes. The same direct frame-to-frame matching procedure used for the linked movies was applied to unlinked movies in terms of the corresponding pixel count of central items and scrambled backgrounds of each image (see https://vimeo.com/megmovieclips for example stimuli).

In each condition every image frame was presented for 700 ms, a duration identified by piloting as being long enough to comprehend the scene or pattern being viewed, and brief enough to minimize saccades and limit fixations to the center of frames. Between each image, “gap” frames of the same duration were inserted, where no image was displayed and which consisted of only a pixelated gray background (see Fig. 1). The pixelation served to mask the visual persistence of the preceding image. Since images were presented in a sequence, the primary function of gaps was to act as a temporal separator so that individual images could be subjected to analysis independently. Gaps also ensured images in unlinked movies were clearly perceived as independent, and the inclusion of gaps in the linked movies ensured close matching. The 16 gaps matched the number of images in each movie clip, and each movie ended with a gap.

Pilot testing of a larger number of stimuli ensured that we only included in the main experiment those patterns movies that were not interpreted as depicting real objects, scenes, or social events. We also confirmed that the gaps between images did not interrupt the naturalistic comprehension of linked movies or their sense of unfolding. During piloting, each individual movie was also rated on: perceived linking, how linked (or disconnected) images appeared to be; and thinking ahead, how much of the time people found themselves thinking about what might happen next. A significant Friedman test for perceived linking (*n* = 7; χ^2^_(3)_ = 18, *p *=* *0.0004) followed by Wilcoxon signed-rank tests found no significant difference between the two linked conditions (*Z* = −0.314, *p *=* *0.753), or between the two unlinked conditions (*Z* = −0.368, *p *=* *0.713). There was, as expected, a significant effect of linking when comparing pictures-linked with pictures-unlinked (*Z *=* *2.371, *p *=* *0.018), and patterns-linked with patterns-unlinked (*Z *=* *2.366, *p *=* *0.018). Similarly, for perceived thinking ahead, a significant Friedman test (χ^2^_(3)_ = 17.735, *p *=* *0.0005) was followed by Wilcoxon signed-rank tests that revealed no significant difference between the two linked conditions (*Z* = −0.169, *p *=* *0.866), or between the two unlinked conditions (*Z* = −0.271, *p *=* *0.786). There was a significant effect of thinking ahead when comparing pictures-linked with pictures-unlinked (*Z *=* *2.371, *p *=* *0.018), and patterns-linked with patterns-unlinked (*Z *=* *2.366, *p *=* *0.018).

### Prescan training

Participants were trained before the MEG scan to ensure familiarity with the different conditions and the rate of presentation of movie frames. These practice movies were not used in the main experiment. Specifically, participants were shown examples and told: “The movie clips are made up of a series of images. Some clips have images that are clearly linked to each other, and some have images that are not linked at all, so the images are completely unrelated to one another. The images can be either pictures with a stick-figure character, or abstract patterns.” For pictures-linked movies, it was explained “…as you can see, there is a stick-figure character doing something. You’ll have noticed that the pictures are all linked together so that the clip tells a story.” For patterns-linked movies it was explained: “…for this type of clip, the patterns are all linked together, so one pattern leads to the next one in the clip. In this example clip the pattern moved outwards at first, then the crosses became larger, and then the circles increased in size, then the pattern changed again. The shape changed a bit step-by-step so that the clip portrays an evolving pattern.” Participants were instructed not to link the images in unlinked movies and to treat each image frame separately when viewing them.

Movies were preceded by one of four visual cues: pictures-linked, patterns-linked, or, for the control conditions, pictures-unlinked and patterns-unlinked (Fig. 1*A*), to advise a participant of the upcoming condition. Cues were provided in advance of each movie so that participants would not be surprised to discover the nature of the clip. Without a cue, the experiment would be poorly controlled since there would most likely be differences across participants in terms of when they registered the clip type during its viewing. This would make it impossible to time-lock processing of the clip to neural activity in a consistent manner across participants. Instead, by using an informative cue, we could be sure that from the very first image frame a participant understood whether the movie was to be composed of linked images or not, and whether these images would depict pictures or patterns.

### Task and procedure

Scripts run in MATLAB R2018a were used to present stimuli and record responses in the MEG scanner. Each trial was preceded by a cue advising of the upcoming condition (e.g., pictures-linked) which was shown for 3000 ms. Each movie was 22,400 ms in duration from the appearance of the first image frame to the end of the final gap frame (Fig. 1*A*). Individual image and gap frames were each 700 ms in duration. Participants then saw a fixation cross for 3000 ms before the next cue. To ensure participants attended to the movies throughout the scanning session, an occasional probe question was included (two trials per condition; Fig. 1*B*). Following the final gap frame of a movie, a novel image was presented (either a picture or a pattern) and participants were asked whether this image fitted well with the movie clip they just saw. Of the two probe trials per condition, one was a “yes” trial (the image was congruent with the movie), and one was a “no” trial (the image was incongruent with the movie).

Given the rate at which frames were presented, we sought to minimize a systematic relationship between spontaneous blinking and stimulus onset. Furthermore, fatigue is known to increase blink duration, which could result in participants missing individual frames, and increase the risk of significant head movement. Consequently, to ensure participants remained alert, the scanning session was split into five blocks each lasting ∼6 min. During breaks between recordings participants were instructed to blink and rest. Each recording block contained eight movie trials where conditions were presented in a randomized order for each participant. Participants were instructed to maintain fixation in the center of frames during the entire trial and to restrict eye movements to between-trial periods.

In summary, movies were visually similar, with one-to-one matching between the two linked and also the two unlinked conditions. Common to all movies was the use of a central item per image, the inclusion of interleaved gap frames, use of simple line illustrations of pictures or patterns in grayscale, all of which were presented at the same frame rate of 1.43 frames per second.

### In-scanner eye tracking and analysis

An Eyelink 1000 Plus (SR Research) eye tracking system with a sampling rate of 2000 Hz was used during MEG scanning to monitor task compliance and record data (x and y coordinates of all fixations) across the full screen. The right eye was used for a 9-point grid calibration, recording and analyses. For some participants the calibration was insufficiently accurate, leaving 16 datasets for eye tracking analyses. The Eyelink Data Viewer (SR Research) was used to examine fixation locations and durations. We used the built-in online data parser of the Eyelink software whereby fixation duration was parsed automatically with fixations exceeding 100 ms. Eye tracking comparisons involving all four conditions were performed to examine where (using group eye fixation heat maps) and for how long (using a two-way repeated measures ANOVA) participants fixated during a 700-ms time window. Our primary focus was on comparing the neural activity evoked during the pictures-linked and patterns-linked conditions. Consequently, the outcome of this comparison directed our subsequent examination of the eye tracking data, meaning that we focused the eye tracking analysis on the specific time windows where differences in the neural data were identified. This allowed us to ascertain whether the neural differences between conditions could have been influenced by oculomotor disparities.

### Postscan surprise memory test

Following the experiment, participants completed a surprise free recall test for the event movies, since the principal aim was to examine the neural differences between pictures-linked and patterns-linked movies. Participants were asked to recall everything they could about what happened in each of these clips, unprompted. If they correctly recalled the simple story, they scored “1” for that clip, otherwise they scored “0.” Specifically, a score of 1 was awarded if all of the following information was provided: a description of the main figure (be it a stick-figure or abstract pattern) and context, and a narrative containing all of the subevents that unfolded. The maximum score per participant and event condition was therefore 10 (as there were 10 movies per condition). Performance for pictures-linked and patterns-linked were compared using a paired-samples *t* test with a statistical threshold of *p *<* *0.05.

### MEG data acquisition

MEG data were acquired using a whole-head 275-channel CTF Omega MEG system within a magnetically shielded room with a sampling rate of 1200 Hz. Participants were scanned in a seated position, with the back of their head resting on the back of the MEG helmet. Head position fiducial coils were attached to the three standard fiducial points (nasion, left and right preauricular) to monitor head position continuously throughout acquisition.

As noted above, we were particularly interested in hippocampal neural activity. The ability of MEG to detect deep sources, including the hippocampus, has been previously debated (Mikuni et al., 1997; Shigeto et al., 2002). While it is inevitable that spatial resolution decreases with depth (Hillebrand and Barnes, 2002), evidence has accumulated to convincingly establish that MEG can indeed localize activity to the hippocampus (Meyer et al., 2017; Pu et al., 2018; Ruzich et al., 2019). This includes during autobiographical memory event retrieval (McCormick et al., 2020), imagination (Barry et al., 2019; Monk et al., 2021), and memory encoding (Crespo-García et al., 2016). Separate fMRI, MEG, and intracranial EEG (iEEG) studies using the same virtual reality paradigm have also revealed similar hippocampal (theta) activity across modalities (Doeller et al., 2008; Kaplan et al., 2012; Bush et al., 2017). Particularly compelling are studies using concurrent MEG and iEEG, where the ground truth is available, that have demonstrated MEG can successfully detect hippocampal activity using beamforming (Crespo-García et al., 2016). We were, therefore, confident that we could record neural activity from the hippocampus.

### MEG data preprocessing

MEG data were preprocessed using SPM12 (www.fil.ion.ucl.ac.uk/spm). Data were high-pass filtered at 1 Hz to eliminate slow drifts in signals from the MEG sensors. A band-stop filter was applied at 48–52 Hz to remove the power line interference, and at 98–102 Hz to remove its first harmonic. Epochs corresponding to each movie cue were defined as −100–1000 ms relative to cue onset. Image frames were defined as −100–700 ms relative to image onset. Gap periods were defined as −100–700 ms relative to gap onset. Epochs were concatenated across trials for each condition, and across scanning sessions. Before the calculation of event-related fields (ERFs), data were first low-pass filtered using a two-pass sixth order Butterworth filter, with a frequency cutoff of 30 Hz. We implemented a broadband approach (1–30 Hz), since the focus of this experiment was evoked activity. Although activity within the theta band (4–8 Hz) is often associated with the hippocampus (Colgin, 2013, 2016), there is also evidence for the role of alpha (9–12 Hz) and beta (13–30 Hz) power in event processing (Hanslmayr and Staudigl, 2014). Following visual inspection of the data, an average of only 0.76 epochs were discarded on the basis of containing eye blinks and muscle artifacts. To baseline-correct, the activity during the first 1000 ms from the onset of the fixation period was averaged and subtracted from each cue, image or gap epoch. The robust average was calculated to obtain an ERF per participant and condition. This averaging method down-weights outliers when computing the average and helps to suppress high-frequency artifacts and minimizes trial rejection (Wager et al., 2005).

### MEG data analyses

Our principal aim was to assess differences between the pictures-linked and patterns-linked conditions since our main interest was in comparing the processing of events built from scenes with those built from non-scenes. In order to make this key comparison, our focus was on the individual image frames that composed the movies. As previously mentioned, gaps were included in the design to provide temporal separation between images, so that brain activity associated with each movie image could be separately examined without interference or leakage from the previous image. Consequently, we explored both the evoked responses to particular images along the time course of the movies and then, as a second step, the likely sources of these responses. These steps are described below.

### ERF analysis

ERFs were analyzed using the FieldTrip toolbox (Oostenveld et al., 2011), implemented in MATLAB R2018a, on the robust averaged data per condition. A targeted sliding window approach was used to examine differences between the two event conditions within salient time windows during movies, namely images 1, 2, 8, and 16. At image 1, only this first single image of a sequence was being viewed; at image 2, there was already the context set by the preceding first image; image 8 represented the mid-point of a sequence; and image 16 was the final image. This approach enabled sampling across a long clip length, while also minimizing multiple comparisons. A number of secondary contrasts involving the premovie cues, the gap frames, and control conditions were also performed to examine whether any differences observed between the two event conditions could be explained by other factors.

We used a non-parametric cluster-based permutation approach for our ERF analyses, a commonly adopted approach that deals with the multidimensional nature of MEG (and EEG) data (see Maris, 2004, 2012; Maris and Oostenveld, 2007). Cluster-based correction addresses both issues of correlation (since electrophysiological responses are necessarily correlated) and multiple comparisons, balanced with maximizing the sensitivity to detect an effect in multidimensional data. The cluster-based permutation approach corrects for multiple comparisons across all MEG channels and time samples across a specific time window. It also controls for the Type I error rate by identifying clusters of significant differences over time and sensors rather than performing separate tests for each sample of time and space. This makes it a particularly powerful approach for MEG/EEG data, and a statistically robust method to determine time windows and sensor locations of effects.

Specifically, each pairwise comparison was performed using the non-parametric cluster-based permutation test, providing a statistical quantification of the sensor-level data while correcting for multiple comparisons across all MEG channels and time samples (Maris and Oostenveld, 2007), across the first 1000 ms of the cue and the entire 700 ms of images and gaps. Cluster-level statistics are the sum of *t* values within each cluster, and this was calculated by taking the maximum cluster-level statistic (positive and negative separately), over 5000 random permutations of the observed data. The obtained *p* value represents the probability under the null hypothesis (no difference between a pair of conditions) of observing a maximum greater or smaller than the observed cluster-level statistics. We report only effects that survived this correction (familywise error, *p *<* *0.05).

We also examined a possible interaction involving all four conditions, using the same cluster-based permutation test, namely on the difference between differences: (pictures-linked – pictures-unlinked) minus (patterns-linked – patterns-unlinked).

### Source reconstruction

Following the sensor-level ERF cluster-based statistical analyses, where we controlled for multiple comparisons over sensors and time-points, we then performed a *post hoc* source reconstruction analysis within the time window already identified at the sensor-level as significant. Source reconstruction, therefore, serves to interrogate the sensor level results further and illustrate the sources of the effect already identified. Consequently, the peaks at the source level are reported without requiring further correction for multiple comparisons over the whole brain (see Gross et al., 2013), as this was already performed at the sensor level. Source reconstruction was performed using the DAiSS toolbox (https://github.com/SPM/DAiSS) included in SPM12. The linearly constrained minimum variance (LCMV) beamformer algorithm (Van Veen et al., 1997) was used to generate maps of power differences between conditions of interest, as informed by the preceding ERF results. For each participant, the covariance matrix was estimated using a common spatial filter for all conditions. For consistency, this was performed within the same broadband frequency spectrum as the ERF analysis (1–30 Hz). Because of the narrow time window of interest (<700 ms), this spatial filter was computed across the entire epoch (−700–700 ms) including both preresponse and postresponse windows. Whole-brain power images per condition and per participant were subsequently generated within only the shorter interval of interest identified by the ERF analysis. Coregistration to MNI space was performed using a 5-mm volumetric grid and was based on nasion, left and right preauricular fiducials. The forward model was computed using a single-shell head model (Nolte, 2003). This resulted in one weight-normalized image per participant within the interval of interest for each condition, that were then smoothed using a 12-mm Gaussian kernel, and a t-contrast was performed at the second level.

## Results

### Behavioral results

Participants correctly identified images as either being congruent or incongruent with the antecedent clip on an average of 93% of probe trials (SD 0.75), confirming they maintained their attention throughout the scanning session.

### Eye tracking results

**Figure 2.**
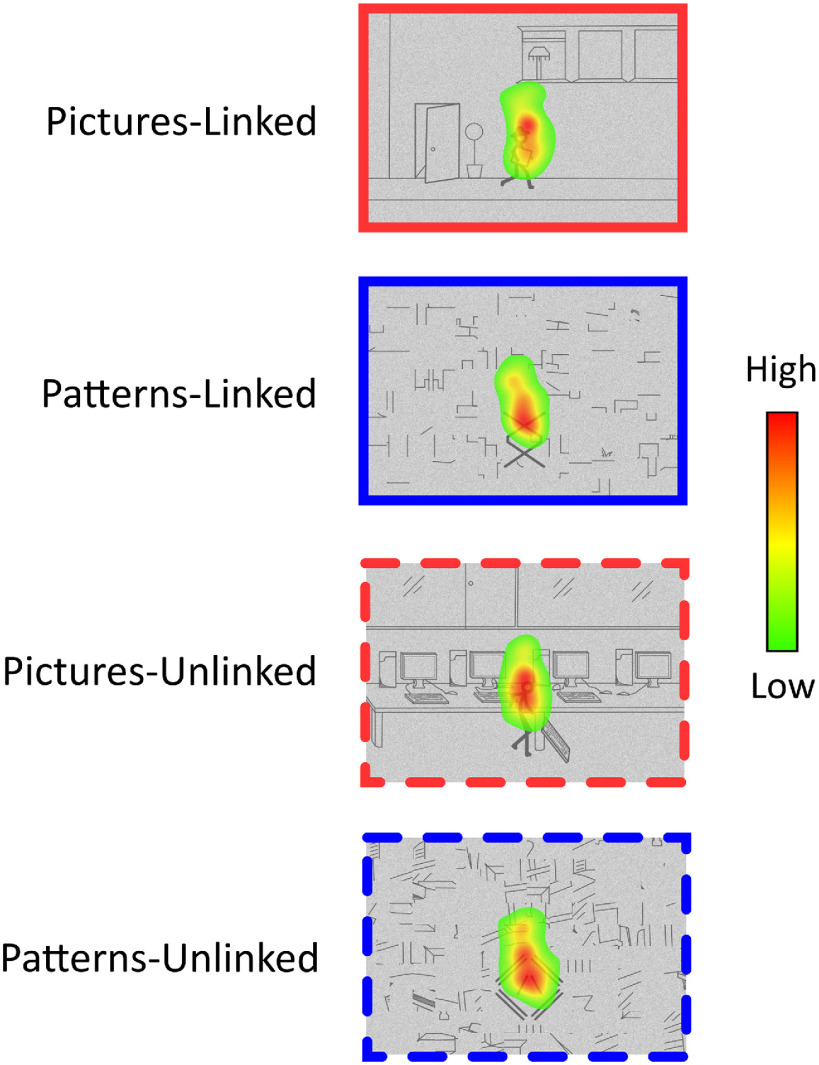
Eye tracking results. Group eye fixation heat maps for each condition during the image 1 time window. Red indicates higher fixation density, and green lower fixation density.

Oculomotor behavior was examined during the time window where ERF analyses showed the only significant difference between the pictures-linked and patterns-linked conditions, which was during the first image. Fixation heat maps revealed the spatial pattern of fixations during image 1 (0–700 ms; Fig. 2) were highly similar across the conditions, and confirmed that participants maintained their focus on the center of the images. No significant difference in fixation count was found between conditions during image 1 (*F*_(3,45)_ = 0.535, *p *=* *0.661), and this included between pictures-linked and patterns-linked (*t*_(15)_ = 0.141, *p *=* *0.889).

### Postscan surprise memory test

After scanning, participants engaged in a surprise free recall memory test for pictures-linked and patterns-linked movies. A paired samples *t* test revealed no significant difference in recall (pictures-linked mean 9.48, SD 1.21; patterns-linked mean 9.24, SD 0.89; *t*_(20)_ = 0.7555, *p *=* *0.459). Participants, therefore, successfully encoded both types of event stimuli, although they were never instructed to do so.

### ERFs

**Figure 3.**
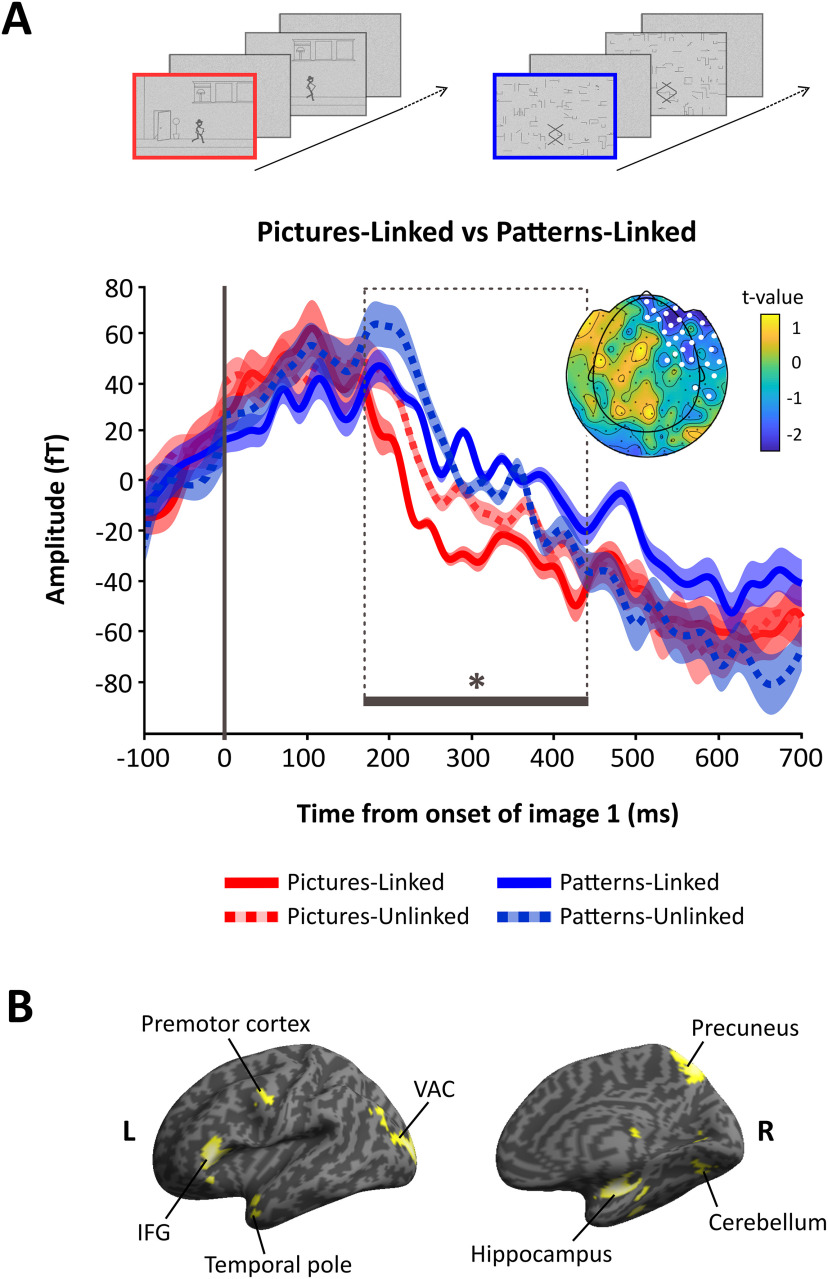
MEG results. ***A***, The ERF analysis revealed a significant difference between pictures-linked (bold red line) and patterns-linked (bold blue line) for first image frame between 178 and 447 ms (*p =* *0.0398), indicated by the dashed line box. Displayed are the grand-averaged ERFs (shading indicates the SEM) for all four conditions, averaged over a right frontotemporal cluster (marked by white dots on the adjacent topoplot) within which the significant difference between pictures-linked and patterns-linked was observed. Displayed to the right of the ERF panel is the topographic distribution of the difference (*t* values), displayed over a helmet layout. Pictures-unlinked is represented by the dashed red line, and patterns-unlinked by the dashed blue line. ***B***, Source reconstruction of evoked activity at image 1 during the 178 to 447ms interval, displayed on a rendered inflated cortical surface, thresholded at *p* <0.005 uncorrected. L, left hemisphere; R, right hemisphere; IFG, inferior frontal gyrus; VAC, visual association cortex.

The primary focus was on comparing the pictures-linked and patterns-linked conditions. We examined this contrast across all time windows of interest, from the cue preceding the movie, to the final image frame, and similarly for the equivalent gap frames. The only significant difference between pictures-linked and patterns-linked conditions was evident at the very first image, involving one negative cluster emerging between 178–447 ms (*p *=* *0.0398) and distributed across right frontotemporal sensors (Fig. 3*A*). This difference could not be because of an effect of image type (i.e., pictures) per se, as no difference was observed at image 1 between pictures-unlinked and patterns-unlinked (*p *=* *0.173). In fact, it is clear from Figure 3*A*, that pictures-linked sits apart from the other three conditions, differing significantly from not only patterns-linked (as already noted), but also patterns-unlinked (*p *=* *0.013), and approaching significance for pictures-unlinked (*p *=* *0.0678). The other three conditions do not differ from one another: this includes patterns-linked and patterns-unlinked (*p *=* *0.3583), and patterns-linked and pictures-unlinked (*p *=* *0.357), showing that the effect found cannot be because of linking per se

This pattern of results, with pictures-linked driving the effect at image 1, suggests that there may be an interaction effect across the four conditions during this image frame. When this was tested formally, we found there was indeed a significant interaction effect at image 1 (*p *=* *0.012; time window = 164–475 ms) encompassing the same time window within which pictures-linked and patterns-linked diverged (see Fig. 3*A*).

### Source reconstruction

We subsequently performed a beamformer analysis on the image 1 pictures-linked versus patterns-linked contrast, restricted to the same time window (178–447 ms) and frequency band (1–30 Hz) within which the significant difference in evoked responses was found. This analysis served to give a better indication of where in the brain this difference originated. The primary peak difference was found in the right hippocampus for pictures-linked relative to patterns-linked (peak *x*, *y*, *z *=* *32, −20, −16; Fig. 3*B*), along with the right precuneus (12, −66, 56), left visual association cortex (−22, −96, 10), left inferior frontal gyrus (−48, 26, 2), left premotor cortex (−48, −2, 38), and right cerebellum (−30, −64, −58).

## Discussion

Unfolding events are central to how we experience the world. In this study we had participants watch dynamic, movie-like events, and compared those built from successively linked scenes (pictures-linked) to those composed of successively linked non-scene patterns (patterns-linked). By using an ERF sliding time window approach to the analysis, we strategically examined image frames across the movies. This novel design allowed a millisecond-by-millisecond examination of the transition from a single image frame to an unfolding event, with a particular interest in hippocampal responses. Only one difference between the closely matched scene and non-scene events emerged, and that was within 178–447 ms of the onset of the first image frame, detectable across frontotemporal sensors. Further probing of this difference using source reconstruction revealed greater engagement of a set of brain regions across parietal, frontal, premotor, and cerebellar cortices, with the largest change in broadband (1–30 Hz) power in the hippocampus during pictures-linked events.

A notable feature of the results is that the only difference between scene and non-scene-based events was during viewing of the first image frame, a point at which an event was yet to unfold. Participants were cued before each trial to inform them which condition was to come, but there was no difference apparent between the two conditions during the cue phase. Rather, the two event types diverged only when an event was initiated. This shows that that the ERF difference found at the first movie image did not merely bleed in from the preceding cue period.

A small number of previous MEG studies have investigated the neural correlates of event processing particularly in the form of autobiographical memory recall (Fuentemilla et al., 2014, 2018; Hebscher et al., 2019, 2020; McCormick et al., 2020). Just one of these studies examined the earliest point of event recall initiation (McCormick et al., 2020) and found that within the first 200 ms of autobiographical event retrieval, the hippocampus was engaged. Another recent MEG study is also relevant. Monk et al. (2021) contrasted the step-by-step building of scene imagery from three successive auditorily-presented object descriptions and an imagined 3D space. This was contrasted with constructing mental images of non-scene arrays that were composed of three objects and an imagined 2D space. They observed a power change in the hippocampus during the initial stage of building scene compared with non-scene imagery. Our finding of an early hippocampal response for the scene-based events aligns with these extant studies.

Why would the difference in hippocampal engagement between scene and non-scene-based events be apparent at the first image frame? One might have expected that it would require events to unfold at least over a couple of image frames for significant changes in neural activity to be evident. However, it may be that the stage was set, so to speak, as soon as the first scene image was viewed. The context of a scene-based event is clearly apparent at that point, and thereafter each additional increment in information provided by subsequent image frames was relatively small. However, the same was true for the non-scene events, and yet the hippocampus was differentially responsive to initial frames of scene-based events. Notably, after the first image frame, the hippocampal response to the subsequent unfolding events was indistinguishable between the two conditions.

Despite the first image frames of pictures-linked and patterns-linked stimuli being composed of the same elements, and both setting a context for an event, it was when the first image resembled the real world that the hippocampal response was elicited. The influence of scene imagery in events is unsurprising given how it mirrors the way in which we experience our surroundings as scene snapshots between blinks and saccades (Clark et al., 2020). Indeed, it has been suggested that one function of the hippocampus may be to support the construction of internal models of the world in the form of scene imagery even during perception (Maguire and Mullally, 2013), and our results are supportive of the link between the hippocampus and scene processing. But what is it about a scene image that provoked the hippocampal response? As noted above, even a single scene can provide a clear indication of the context, and hippocampal engagement may reflect this context being registered.

Further insight may be gained by looking at the pictures-unlinked control condition. In both the pictures-linked condition and the pictures-unlinked control condition a single image could provide a clear indication of a context. Nevertheless, there was a (near-significant) ERF difference between these two conditions at the point of the first image. This suggests that it may be more than the registration of the real-world context that is the influential factor, as contexts were present in both conditions. In the pictures-linked condition, a participant knew that the context depicted in the first image was going to endure for the entire clip, because the cue preceding each clip advised of the upcoming condition. Similarly, they knew that each image in the pictures-unlinked condition related to that image alone, and would not endure across the clip. Consequently, it may be that for the first image in a pictures-linked movie, the context is registered, perhaps a relevant scene template or schema is activated fully (Gilboa and Marlatte, 2017), and then used to help link each image across the sequence. In contrast, the first image in a pictures-unlinked clip may be limited to just registering the context.

Our finding of a very early hippocampal response to unfolding scene-based events differs from fMRI studies of movie viewing that found the hippocampus responded later, toward the offset of events, with the speculation that this reflected event replay, aiding memory consolidation (Ben-Yakov and Dudai, 2011; Baldassano et al., 2017; for review, see Griffiths and Fuentemilla, 2020). There are several differences between our study and this previous work. For instance, the latter typically involved explicit memory encoding, participants knew they would be tested afterward, and this may have influenced hippocampal engagement toward the end of events if memory rehearsal occurred. By contrast, our task had no memory demands, although excellent incidental encoding took place. In addition, our study was not designed to assess event boundaries; indeed, our two conditions were very highly matched in terms of event structure, which may have precluded boundary-related findings. Prior studies also used fMRI, which is blind to rapid, phasic neuronal activity, given the slow nature of the hemodynamic response. The few EEG studies that have examined memory encoding using movies were conducted at the sensor level (Silva et al., 2019), and did not source localize responses to specific brain structures. Further MEG studies in the future would be particularly helpful in extending event, and event boundary, research to characterize more precisely the temporal dynamics of hippocampal activity.

Beyond the hippocampus, our results also revealed the involvement of a broader set of brain regions associated with pictures-linked more so than patterns-linked movies, namely, the posterior parietal, inferior frontal, premotor, and cerebellar cortices. Consideration of these areas may shed further light on differences between the two conditions. These brain areas have been identified in numerous studies as part of a network that processes biological motion and the anticipation of incoming intentional movement (Battelli et al., 2003; Rizzolatti and Craighero, 2004; Saygin et al., 2004; Fraiman et al., 2014). In particular, this has been observed in the context of point-light displays, in which a small number of moving lights (e.g., at the joints of a moving person) are sufficient to interpret this as behavior (e.g., dancing). The pictures-linked events were highly simplified portrayals of activities depicted by stick-figures, lines and circles to create simple scenes. Although 2D drawings, they evoked 3D unfolding events of real-world activities that were easily grasped by participants. Scene-based and pattern-based evolving stimuli may have been processed differently because abstract patterns were not perceived as intentional, biological stimuli, while participants could automatically infer the actions performed in scene-based events, even as early as the first image frame. Indeed, through piloting we sought to exclude patterns that consistently evoked the sense of biological motion. The success of our efforts was reflected in the descriptions provided by participants in the postscan memory test. For example, elements of a patterns-linked movie showing three overlapping diamond shapes was described as “diamond shapes gradually expanded outwards, then rotated clockwise,” while pictures-linked movies were typically described in terms of the intentionality of the stick-figure.

Biological motion is often related to theory of mind. Could theory of mind explain the ERF differences between the pictures-linked and patterns-linked conditions? We feel this is unlikely given that brain areas typically engaged by theory of mind did not emerge in the analyses. Moreover, while biological motion perception appears to relate to some aspects of theory of mind, they are not equivalent constructs (Rice et al., 2016; Meinhardt-Injac et al., 2018). For example, people with theory of mind deficits (e.g., in the context of autism) may demonstrate deficits in the perception of biological motion relative to controls but this may depend on whether emotional state information is required (Todorova et al., 2019). Whether there is a common neural circuitry underlying biological motion and theory of mind remains unclear. It is likely that the ability to perceive biological motion is required to make social judgements, but it is not the sole component of theory of mind processing (Fitzpatrick et al., 2018). We suggest that our simple, emotionally neutral event movies did not necessarily induce theory of mind processes and, consequently, engagement of brain areas associated with theory of mind was not increased for pictures-linked stimuli.

What other alternative explanations might there be for the hippocampal difference between pictures-linked and patterns-linked movies? It could be argued that the effect of pictures-linked was simply the result of scene processing per se. If this was the case, then a difference ought to have been observed between the pictures-unlinked and patterns-unlinked conditions, since the hippocampus is known to respond strongly to scenes relative to non-scene stimuli (Hassabis et al., 2007a; Graham et al., 2010; Zeidman et al., 2015; Hodgetts et al., 2016; Monk et al., 2021); however, no difference was apparent. This suggests that the type of image alone cannot explain the observed hippocampal effect. Another possibility is that linking or sequencing accounts for the finding. However, linking and unfolding sequences were features of both pictures-linked and patterns-linked, and so this factor cannot easily explain the change in hippocampal power. In addition, no significant differences between any other pairs of conditions, including between patterns-linked and patterns-unlinked, and patterns-linked and pictures-unlinked were evident, suggesting the effect was not solely explained by the linking of images. It seems that the hippocampus responded to the first scene image only when the expectation was that this picture was the start of a linked, unfolding event, as reflected in the image type by linking interaction that we observed.

Despite the measures taken to closely match event stimuli in terms of their sense of unfolding, scenes could simply be more engaging or predictable than pattern-based events. If so, then one might have expected event memory to differ in the surprise postscan test, but it did not, and both types of movie clips were easily recollected as clear narratives. We might also have expected to observe differences in oculomotor behavior, but none were evident, also an indication of similar attentional processes for the two conditions. Consequently, we can conclude that the neural difference identified between the two conditions was not because of a large divergence in encoding success. However, we acknowledge that memory differences might emerge with more complex stimuli. Furthermore, events were very well matched in terms of the number of subevents, and their evolving nature as reflected in the highly similar ratings for “linking” and “thinking ahead” measures during piloting. It also seems unlikely that the difference between the two event types can be explained by working memory load. If pictures-linked movies were easier to hold in mind, while patterns-linked were more effortful to process, we would have expected this to be reflected at later points in the movie clips, as memory load increased, but no such effect was apparent.

In summary, this MEG study revealed very early hippocampal engagement associated with the viewing of events built from scenes, over and above highly matched evolving sequences built from non-scene imagery. Together with the hippocampus, the involvement of other brain regions, including posterior parietal, inferior frontal, premotor, and cerebellar cortices, may reflect the processing of biologically-relevant information, which typifies the scene-rich episodes we encounter in our daily lives.

## Synthesis

Reviewing Editor: Ifat Levy, Yale University School of Medicine Decisions are customarily a result of the Reviewing Editor and the peer reviewers coming together and discussing their recommendations until a consensus is reached. When revisions are invited, a fact-based synthesis statement explaining their decision and outlining what is needed to prepare a revision will be listed below. The following reviewer(s) agreed to reveal their identity: Yaara Yeshurun.

The authors have adequately addressed reviewers’ comments. The manuscript has improved and the text is clearer.

## Author Response

Revision of eN-NWR-0099-21

Dear Dr Levy,

Thank you for sending a synthesis of the Reviewers’ comments on the above-referenced manuscript and for inviting a revised version. We are delighted that you and the Reviewers found the work potentially interesting, and are grateful for the constructive advice on how to improve the manuscript. We have implemented the recommended revisions, which include fleshing out the Introduction and Discussion, and providing substantially more detail in the Materials and Methods to increase clarity. We believe these additions strengthen the paper. Responses to the specific points are detailed below.

Yours sincerely,

The Authors

In this study, the authors used MEG to test the role of the hippocampus in linking between scenes to generate seamless events, as they unfold over time. They report increased hippocampal activation during the first image of linked scene-based events, compared to the first image of unlinked scene-based events, or linked non-scene events. The authors interpret these results as suggesting that the hippocampus is involved in setting the scene very early on.

The paper poses an interesting question, and the experimental design is elegant and has the potential of providing a novel insight into the dynamics of the brain response while linking information to generate a coherent event.

Thank you.

There are, however, serious issues with the analysis and results, as detailed below.

- The definition of "events" in the context of this study is not clear. Are these individual images, entire narrative/story, or the segments/scenes within that larger story (e.g. Silva et al., 2019), or something else? What is the difference between "event" and "sub-event"? The link to example stimuli is helpful, but does not clarify this question.

We apologize for any ambiguity regarding the nature of the event stimuli. In the current study, an event was defined as ‘a dynamic, unfolding sequence of actions that could be described in a story-like narrative’. Each event was a self-contained vignette that lasted 22.4 seconds; an event was not part of a larger story. Each event was composed of a series of individual images. These individual images were linked such that each image led on to the next, thereby depicting an activity that unfolded over the 22.4 seconds. Put another way, the events in this study were digital versions of a flip-book, composed of individual images which, when presented as a sequence, showed the progression of an activity. The insertion of ‘gap’ frames - see the example stimuli provided here: https://vimeo.com/megmovieclips - helped to further emphasise the flip-book nature of the stimuli. For example, one Pictures-Linked movie (available via the web link) is composed of 16 individual static images which when played in sequence depict a stick-figure skate-boarding through a city scene.

The activity depicted in an event (e.g. skate-boarding) can be subdivided into steps. When we describe an event, we can easily provide a step-by-step narrative of what happened; participants were also able to do this after viewing event movies in the current experiment. For example, for the event where the overall activity was skate-boarding, the individual steps included: (1) the stick-figure skate-boards over a ramp, (2) lands on the ground, (3) skate-boards on the ground past some shopping carts, and (4) steps off the skate-board and picks it up. Each of these steps can be described as a ‘sub-event’, and were {greater than or equal to}1 second in duration.

Of note, while some events, such as that involving skate-boarding, reflected the real world, other events depicted abstract patterns whereby incremental changes image-to-image showed a progression of activity that connected every image frame over the course of the 22.4 second clips - Patterns-Linked. Each of these events was also self-contained and not part of a larger narrative. Moreover, each of these events also contained sub-events. For the example provided here: https://vimeo.com/megmovieclips, (1) three diamond shapes are nested within each other, (2) the three diamond shapes start to separate out, (3) the three diamonds become separate and the lines comprising the diamonds become thicker, and (4) the diamond shapes all rotate to the right.

The two event conditions were contrasted with stimuli where images within a 22.4 second clip were not related to each and so could not be regarded as an event. For example, in one Pictures-Unlinked movie (see web link), the first image shows a stick-figure in an internet café, the next image shows a different stick-figure in a trailer park, and each of the remaining images are similarly unrelated to one another. This is also the case for the Patterns-Unlinked condition, where each image showed a distinct abstract shape, with the shapes image-to-image being unrelated to each other.

In the revised manuscript we have elaborated further on the nature of the event stimuli to better explain what ‘event’ means in the context of the current study. Specifically:

Introduction

(p. 2)

Here we defined an event as a dynamic, unfolding sequence of actions that could be described in a story-like narrative.

(p. 3)

...To do this we created a set of short, simple cartoon-like movies each of which depicted an event. Each event was a self-contained vignette, and was composed of a series of individual images. These individual images were linked such that each image led on to the next, thereby depicting an activity that unfolded over 22.4 seconds. In essence, the events were digital versions of a flip-book, composed of individual images which, when presented as a sequence, showed the progression of an activity. We devised two types of movie events. In one, each image frame within a movie was a simple scene reflecting the real world, while in the other condition each image frame comprised abstract shapes. The two event types were visually very similar, and both involved unfolding sequences of individual image frames that could be described in a story-like narrative...

Materials and Methods

(pp. 4-5)

Short visual movies of events were created by hand using the animation program Stykz 1.0.2 (https://www.stykz.net), each composed of 16 individually drawn image frames presented in a sequence. Each of these 16-image movies lasted 22.4 seconds. Each event was self-contained and was not part of a larger story....

(p. 5)

The activity depicted in an event could be subdivided into steps. For example, for the event where the overall activity was skate-boarding (see https://vimeo.com/megmovieclips), the individual steps included: (1) the stick-figure skate-boards over a ramp, (2) lands on the ground, (3) skate-boards on the ground past some shopping carts, and (4) steps off the skate-board and picks it up. Each of these steps, or ‘sub-events’, were {greater than or equal to}1 second in duration. Each of the Patterns-Linked events also contained sub-events. For the example provided here: https://vimeo.com/megmovieclips, (1) three diamond shapes are nested within each other, (2) the three diamond shapes start to separate out, (3) the three diamonds become separate and the lines comprising the diamonds become thicker, and (4) the diamond shapes all rotate to the right.

(p. 6)

There were two control conditions (Figure 1A, lower two panels), each with ten movie clips. Each movie was composed of a series of unique and separate unrelated image frames such that no evolving event could be conceived. Pictures-Unlinked movies contained separate scenes for each image frame, and in total there were 160 unique scenes, twenty different stick-figures and 152 unique objects. For example, in one Pictures-Unlinked movie (see web link), the first image shows a stick-figure in an internet café, the next image shows a different stick-figure in a trailer park, and each of the remaining images are similarly unrelated to one another...

- What does it mean that "single snapshot images transition into unfolding events" (introduction)? It is not at all clear that the authors show this in this paper.

Most of us perceive the world as a series of visual snapshots that are punctuated by eye blinks and saccades. How these separate images become linked together, such that we have a sense of the seamless unfolding of events is unclear. This is the question we sought to address in the current study. To do this we created event stimuli each of which comprised individual images that when viewed sequentially depicted an event. By leveraging the high temporal resolution of MEG, we could examine the neural activity associated with each image in an evolving event sequence. In this way we could speak to the research question. We now clarify this point in the revised manuscript:

Introduction

(p. 4).

The two event types were visually very similar, and both involved unfolding sequences of individual image frames that could be described in a story-like narrative. By leveraging the high temporal resolution of MEG, we could examine each image, allowing us to better understand how a sequence of separate images evolves neurally to give rise to the experience of a seamless event...

- Eye tracking results are presented, but the method of processing and analysis used to reach these results is never mentioned in the materials and methods section.

We have now added more methodological details regarding the eye tracking:

Materials and Methods

(pp. 9-10)

An Eyelink 1000 Plus (SR Research) eye tracking system with a sampling rate of 2000 Hz was used during MEG scanning to monitor task compliance and record data (x and y coordinates of all fixations) across the full screen. The right eye was used for a 9-point grid calibration, recording and analyses. For some participants the calibration was insufficiently accurate, leaving 16 data sets for eye tracking analyses. The Eyelink Data Viewer (SR Research) was used to examine fixation locations and durations. We used the built-in online data parser of the Eyelink software whereby fixation duration was parsed automatically with fixations exceeding 100ms. Eye tracking comparisons involving all four conditions were performed to examine where (using group eye fixation heat maps) and for how long (using a two-way repeated measures ANOVA) participants fixated during a 700 ms time window. Our primary focus was on comparing the neural activity evoked during the Pictures-Linked and Patterns-Linked conditions. Consequently, the outcome of this comparison directed our subsequent examination of the eye tracking data, meaning that we focused the eye tracking analysis on the specific time windows where differences in the neural data were identified. This allowed us to ascertain whether the neural differences between conditions could have been influenced by oculomotor disparities.

Why did you only analyze the eye tracking for image 1 of the sequences?

Our primary focus was on comparing the neural activity evoked during the Pictures-Linked and Patterns-Linked conditions using event-related field (ERF) analyses. Consequently, the outcome of this comparison directed our subsequent examination of the eye tracking data. The ERF analysis showed that the two conditions differed significantly only at the first image in a movie sequence. Therefore, we sought to ascertain whether this neural difference between Pictures-Linked and Patterns-Linked could have been influenced by oculomotor differences during this time window specifically. We have now added a further clarification in the revised manuscript to explain the principle behind our approach to the eye tracking analysis:

Materials and Methods

(p. 10)

Our primary focus was on comparing the neural activity evoked during the Pictures-Linked and Patterns-Linked conditions. Consequently, the outcome of this comparison directed our subsequent examination of the eye tracking data, meaning that we focused the eye tracking analysis on the specific time windows where differences in the neural data were identified. This allowed us to ascertain whether the neural differences between conditions could have been influenced by oculomotor disparities.

- Some additional methodological details are not clear: why was each movie preceded by the label of which condition it was?

Each trial was preceded by a cue in order to advise the participant of the upcoming condition (whether it was Pictures-Linked, Patterns-Linked, or one of the control conditions). In the revised manuscript, we now clarify that a cue was provided in advance of a movie so that participants would not be surprised to discover the nature of the clip. Without a cue, the experiment would be poorly controlled since there would most likely be differences across participants in terms of when they registered the clip type during its viewing. This would make it impossible to time-lock processing of the clip to neural activity in a consistent manner across participants. Instead, by using an informative cue, we could be sure that from the very first image frame a participant understood whether the movie was to be composed of linked images or not, and whether these images would depict pictures or patterns.

Materials and Methods

(p. 8)

Movies were preceded by one of four visual cues - Pictures-Linked, Patterns-Linked or, for the control conditions, Pictures-Unlinked and Patterns-Unlinked (Figure 1A) - in order to advise a participant of the upcoming condition. Cues were provided in advance of each movie so that participants would not be surprised to discover the nature of the clip. Without a cue, the experiment would be poorly controlled since there would most likely be differences across participants in terms of when they registered the clip type during its viewing. This would make it impossible to time-lock processing of the clip to neural activity in a consistent manner across participants. Instead, by using an informative cue, we could be sure that from the very first image frame a participant understood whether the movie was to be composed of linked images or not, and whether these images would depict pictures or patterns.

It is not clear what the order of movie presentations was.

As described in the Materials and Methods section (p. 9), the order of conditions, and so the movie presentations, was randomised across each scanning block and participant.

- In the post-scan memory test - how did participants describe the pattern videos? Was there a narrative to recall? It seems that it would be much easier to recall the picture-linked story than any other story, and probably this event’s memory would be much more detailed. Thus, a score of 0 or 1 for memory does not seems like a sensitive enough measure. How was the recall graded? Generating a more informative measure for the post-scan memory would allow for additional analysis that could support the authors’ main argument. For example, if the hippocampal activity on the first frame in the pictures-linked video is indeed related to building the event from the scenes, it would be interesting to test whether hippocampal activation during the first frame of the video predicts post-scan memory for the video. This analysis could be done only after a more sensitive measure of post-scan memory recall would be generated. Such a measure could also support the authors’ following claim in the discussion: "Despite the measures taken to closely match event stimuli in terms of their sense of unfolding, scenes could simply be more engaging or predictable than pattern-based events. If so, then one might have expected event memory to differ in the surprise post-scan test, but it did not, and both types of movie clips were easily recollected as clear narratives."

Thank you for raising this interesting point. The event movie clips were very simple and brief (see examples here: https://vimeo.com/megmovieclips). Consequently, it is challenging to devise a more nuanced and detailed memory test. A score of 1 was only awarded if the following information was provided: a description of the main figure (be it a stick-figure or abstract pattern) and context, and a narrative containing all of the sub-events that unfolded. Even though the participants were not instructed to memorize the movies, the near-ceiling scores for both event conditions on the surprise post-MEG scan memory test showed that they did so with ease. Consequently, we can conclude that the neural difference identified between the two conditions was not due to a large divergence in encoding success. However, in the revised manuscript we now acknowledge that memory differences might emerge with more complex stimuli, as well as providing additional details about the scoring in the Materials and Methods section. Specifically:

Materials and Methods

(p. 10)

Following the experiment, participants completed a surprise free recall test for the event movies, since the principal aim was to examine the neural differences between Pictures-Linked and Patterns-Linked movies. Participants were asked to recall everything they could about what happened in each of these clips, unprompted. If they correctly recalled the simple story, they scored ‘1’ for that clip, otherwise they scored ’0’. Specifically, a score of 1 was awarded if all of the following information was provided: a description of the main figure (be it a stick-figure or abstract pattern) and context, and a narrative containing all of the sub-events that unfolded. The maximum score per participant and event condition was therefore 10 (as there were 10 movies per condition). Performance for Pictures-Linked and Patterns-Linked were compared using a paired-samples t-test with a statistical threshold of p < 0.05.

Discussion

(pp. 22-23)

Despite the measures taken to closely match event stimuli in terms of their sense of unfolding, scenes could simply be more engaging or predictable than pattern-based events. If so, then one might have expected event memory to differ in the surprise post-scan test, but it did not, and both types of movie clips were easily recollected as clear narratives. We might also have expected to observe differences in oculomotor behavior, but none were evident, also an indication of similar attentional processes for the two conditions. Consequently, we can conclude that the neural difference identified between the two conditions was not due to a large divergence in encoding success. However, we acknowledge that memory differences might emerge with more complex stimuli.

- It is not clear how the difference between Patterns-Linked and Patterns-Unlinked can approach a significant difference at the very first image. Why would there be a difference between the conditions when at the point of the first image the subject doesn’t know whether or not the following images are going to be linked or unlinked? This is important, because the authors’ main argument hinges on this difference - otherwise, it is not clear that the finding is about event processing, rather than a difference in the processing of a single image, which happens to be the first in a series of images.

As we report in the Results section, there was no significant difference (p = 0.3583) in terms of the evoked neural response to the first image when Patterns-Linked and Patterns-Unlinked stimuli were compared.

We wonder if the Reviewer in fact meant to ask about the comparison between Pictures-Linked and Pictures-Unlinked, as this is a result that approached significance (p = 0.0678). As noted in our response to a previous point above, a cue preceded each trial that informed the participant about the type of movie in the upcoming trial. Therefore, a participant knew before the first image appeared whether a movie would depict an event (e.g. Pictures-Linked) or unrelated images (e.g. Picture-Unlinked). This means that when the first image appeared, the participant could set the context for the rest of the images (and so the event) in the Pictures-Linked condition, but this was not possible in the Pictures-Unlinked condition.

Our significant interaction result showed that the neural differences were driven by the Pictures-Linked condition, suggesting that it was the combination of images that reflected the real-world and their linking into an event that was key. By contrast, images that reflected the real-world alone (Pictures-Unlinked) or linking per se (as was present in the Patterns-Linked condition) were not sufficient to provoke changes in neural activity.

We consider this issue further, and that of the cues, in responses to later related comments.

- In their main analysis, the authors had performed at least 5(images) * 4(video’s conditions) comparisons, across MEG channels and time samples (comparing the activation in each channel, at each time point during a 700 ms period). These are many comparisons, and it is not clear how the non-parametric cluster-based permutation test corrects for them. What did the authors permute in order to generate the null distribution?

Our primary ERF analyses were performed at images 1, 2, 8, and 16 (4 time-points) between Pictures-Linked and Patterns-Linked (2 conditions). Secondary analyses were subsequently performed at the equivalent cues and gap frames, as were contrasts involving the control conditions to examine whether any differences observed between the two main event conditions could be explained by other factors. As the Reviewer correctly observed, when many comparisons are performed in a statistical test a correction for multiple comparisons is imperative. For this reason, we chose a non-parametric cluster-based permutation approach for our ERF analyses, which deals with the multidimensional nature of MEG (and EEG) data in the most straightforward way, and is a commonly adopted approach to deal with this type of data (see Maris, 2004; Maris, 2012; Maris and Oostenveld, 2007). Cluster-based correction addresses both issues of correlation (since electrophysiological responses are necessarily correlated) and multiple comparisons, balanced with maximising the sentivity to detect an effect in multidimensional data. The cluster-based permutation approach corrects for multiple comparisons across all MEG channels and time samples across a specific time window. It also controls for the Type I error rate by identifying clusters of significant differences over time and sensors rather than performing separate tests for each sample of time and space. This makes it a particularly powerful approach for MEG/EEG data, and a statistically robust method to determine time windows and sensor locations of effects. We report only effects that survived this correction. In the revised manuscript, we now provide more information on the non-parametric cluster-based permutation approach, including what was permuted:

Materials and Methods

(pp. 13-14)

ERFs were analyzed using the FieldTrip toolbox (Oostenveld et al., 2011), implemented in Matlab R2018a, on the robust averaged data per condition. A targeted sliding window approach was used to examine differences between the two event conditions within salient time windows during movies, namely images 1, 2, 8, and 16. At image 1, only this first single image of a sequence was being viewed; at image 2, there was already the context set by the preceding first image; image 8 represented the mid-point of a sequence; and image 16 was the final image. This approach enabled sampling across a long clip length, whilst also minimizing multiple comparisons. A number of secondary contrasts involving the pre-movie cues, the gap frames, and control conditions were also performed in order to examine whether any differences observed between the two event conditions could be explained by other factors.

We used a non-parametric cluster-based permutation approach for our ERF analyses, a commonly adopted approach that deals with the multidimensional nature of MEG (and EEG) data (see Maris, 2004; Maris, 2012; Maris and Oostenveld, 2007). Cluster-based correction addresses both issues of correlation (since electrophysiological responses are necessarily correlated) and multiple comparisons, balanced with maximising the sentivity to detect an effect in multidimensional data. The cluster-based permutation approach corrects for multiple comparisons across all MEG channels and time samples across a specific time window. It also controls for the Type I error rate by identifying clusters of significant differences over time and sensors rather than performing separate tests for each sample of time and space. This makes it a particularly powerful approach for MEG/EEG data, and a statistically robust method to determine time windows and sensor locations of effects.

Specifically, each pairwise comparison was performed using the non-parametric cluster-based permutation test, providing a statistical quantification of the sensor-level data while correcting for multiple comparisons across all MEG channels and time samples (Maris and Oostenveld, 2007), across the first 1000 ms of the cue and the entire 700 ms of images and gaps. Cluster-level statistics are the sum of t-values within each cluster, and this was calculated by taking the maximum cluster-level statistic (positive and negative separately), over 5000 random permutations of the observed data. The obtained p-value represents the probability under the null hypothesis (no difference between a pair of conditions) of observing a maximum greater or smaller than the observed cluster-level statistics. We report only effects that survived this correction (FWE, p < 0.05).

We also examined a possible interaction involving all four conditions, using the same cluster-based permutation test, namely on the difference between differences: (Pictures-Linked - Pictures-Unlinked) minus (Patterns-Linked - Patterns-Unlinked).

It was also not clear whether there was correction for multiple comparisons in the source reconstruction analysis.

Following the sensor-level ERF cluster-based statistical analyses, where we controlled for multiple comparisons over sensors and time-points and detected a significant effect, it is typical to then perform a post-hoc source reconstruction analysis within the time window already identified at the sensor-level as significant. Source reconstruction therefore serves to interrogate the sensor level results further and illustrate the sources of the effect already identified. Consequently, it is acceptable to report the peaks at the source level without requiring further correction for multiple comparisons over the whole brain (see Gross et al., 2013), as this was already performed at the sensor level. We now further clarify the purpose of source reconstruction in the revised manuscript:

Materials and Methods

(p. 14)

Following the sensor-level ERF cluster-based statistical analyses, where we controlled for multiple comparisons over sensors and time-points, we then performed a post-hoc source reconstruction analysis within the time window already identified at the sensor-level as significant. Source reconstruction, therefore, serves to interrogate the sensor level results further and illustrate the sources of the effect already identified. Consequently, the peaks at the source level are reported without requiring further correction for multiple comparisons over the whole brain (see Gross et al., 2013), as this was already performed at the sensor level. Source reconstruction was performed using the DAiSS toolbox (https://github.com/SPM/DAiSS) included in SPM12...

- The authors claim that the hippocampus is responsible for what they describe as "event processing." MEG, however, is a surface-based technique for assessing the temporal dynamics of brain activity, and there is inherently no perfect inverse solution to definitively localize surface-based activity. The authors can therefore only speculate that the hippocampus is involved in the processing described in the paper.

While it is inevitable that spatial resolution decreases with depth (Hillebrand and Barnes, 2002), evidence has accumulated to convincingly establish that MEG can indeed localize activity to the hippocampus (Meyer et al., 2017; Pu et al., 2018; Ruzich et al., 2019). This includes during autobiographical memory event retrieval (McCormick et al., 2020), imagination (Barry et al., 2019; Monk et al., 2021), and memory encoding (Crespo-García et al., 2016). Separate fMRI, MEG, and intracranial EEG (iEEG) studies using the same virtual reality paradigm have also revealed similar hippocampal (theta) activity across modalities (Doeller et al., 2008; Kaplan et al., 2012; Bush et al., 2017). Particularly compelling are studies using concurrent MEG and iEEG, where the ground truth is available, that have demonstrated MEG can successfully detect hippocampal activity using beamforming (Crespo-García et al., 2016). We are, therefore, confident about the interpretability of our results. In the revised manuscript we now explicitly address the issue of MEG’s ability to detect deep sources:

Materials and Methods

(p. 11)

As noted above, we were particularly interested in hippocampal neural activity. The ability of MEG to detect deep sources, including the hippocampus, has been previously debated (Mikuni et al., 1997; Shigeto et al., 2002). While it is inevitable that spatial resolution decreases with depth (Hillebrand and Barnes, 2002), evidence has accumulated to convincingly establish that MEG can indeed localize activity to the hippocampus (Meyer et al., 2017; Pu et al., 2018; Ruzich et al., 2019). This includes during autobiographical memory event retrieval (McCormick et al., 2020), imagination (Barry et al., 2019; Monk et al., 2021), and memory encoding (Crespo-García et al., 2016). Separate fMRI, MEG, and intracranial EEG (iEEG) studies using the same virtual reality paradigm have also revealed similar hippocampal (theta) activity across modalities (Doeller et al., 2008; Kaplan et al., 2012; Bush et al., 2017). Particularly compelling are studies using concurrent MEG and iEEG, where the ground truth is available, that have demonstrated MEG can successfully detect hippocampal activity using beamforming (Crespo-García et al., 2016). We were, therefore, confident that we could record neural activity from the hippocampus.

Furthermore, if the source localization techniques are to be believed, then the authors found many more brain regions in addition to the hippocampus that were associated with the effect. They should therefore tone down their statement that the responses are due to the hippocampus.

As noted in the Introduction, we were particularly interested in neural responses from the hippocampus, and while other brain regions were also localized by the source reconstruction, the greatest difference in activity between the two conditions of interest was found in the hippocampus. In the Discussion we considered a number of these regions and why they might have been engaged. In the revised manuscript we now signpost this further:

Abstract

(p 1)

Further probing of this difference using source reconstruction revealed greater engagement of a set of brain regions across parietal, frontal, premotor, and cerebellar cortices, with the largest change in broadband (1-30 Hz) power in the hippocampus during scene-based movie events.

Discussion

(p. 17)

Further probing of this difference using source reconstruction revealed greater engagement of a set of brain regions across parietal, frontal, premotor, and cerebellar cortices, with the largest change in broadband (1-30 Hz) power in the hippocampus during Pictures-Linked events.

(p. 21)

Beyond the hippocampus, our results also revealed the involvement of a broader set of brain regions associated with Pictures-Linked more so than Patterns-Linked movies, namely, the posterior parietal, inferior frontal, premotor, and cerebellar cortices. Consideration of these areas may shed further light on differences between the two conditions. These brain areas have been identified in numerous studies as part of a network that processes biological motion and the anticipation of incoming intentional movement (Battelli et al., 2003; Rizzolatti and Craighero, 2004; Saygin et al., 2004; Fraiman et al., 2014). In particular, this has been observed in the context of point-light displays, in which a small number of moving lights (e.g. at the joints of a moving person) are sufficient to interpret this as behavior (e.g. dancing). The Pictures-Linked events were highly simplified portrayals of activities depicted by stick-figures, lines and circles to create simple scenes. Although 2D drawings, they evoked 3D unfolding events of real-world activities that were easily grasped by participants. Scene- and pattern-based evolving stimuli may have been processed differently because abstract patterns were not perceived as intentional, biological stimuli, while participants could automatically infer the actions performed in scene-based events, even as early as the first image frame. Indeed, through piloting we sought to exclude patterns that consistently evoked the sense of biological motion. The success of our efforts was reflected in the descriptions provided by participants in the post-scan memory test. For example, elements of a Patterns-Linked movie showing three overlapping diamond shapes was described as ’diamond shapes gradually expanded outwards, then rotated clockwise’, while Pictures-Linked movies were typically described in terms of the intentionality of the stick-figure...

- In the discussion (Pg. 19), the authors suggest that "As noted above, even a single scene can provide a clear indication of the context, and hippocampal engagement may reflect this being registered, perhaps based on well-established templates or schemas (Gilboa and Marlatte, 2017)." However, the first scene of the unlinked story also provides a clear indication of context, that should elicit specific templates and schemas, and there was no increased hippocampal activity for the first scene of the unlinked story.

Thank you for highlighting this interesting point. It is indeed the case that in both the Pictures-Linked and the Pictures-Unlinked conditions a single image could provide a clear indication of the context. Nevertheless, there was a (near-significant) ERF difference between the two conditions at the point of the first image. In the Pictures-Linked condition, the participant knew that the context depicted in the first image was going to endure for the entire clip, because the cue preceding each clip advised of the upcoming condition. Similarly, they knew that each image in the Pictures-Unlinked condition related to that image alone, and would not endure across the clip. Consequently, it may be that for the first image in a Pictures-Linked movie, the context is registered, a relevant template or schema is activated fully, and then used to help link each image across the sequence. In contrast, the first image in a Pictures-Unlinked clip may be limited to just registering the context. We now elaborate on this point in the revised manuscript:

Discussion

(pp. 19-20)

Further insight may be gained by looking at the Pictures-Unlinked control condition. In both the Pictures-Linked condition and the Pictures-Unlinked control condition a single image could provide a clear indication of a context. Nevertheless, there was a (near-significant) ERF difference between these two conditions at the point of the first image. This suggests that it may be more than the registration of the real-world context that is the influential factor, as contexts were present in both conditions. In the Pictures-Linked condition, a participant knew that the context depicted in the first image was going to endure for the entire clip, because the cue preceding each clip advised of the upcoming condition. Similarly, they knew that each image in the Pictures-Unlinked condition related to that image alone, and would not endure across the clip. Consequently, it may be that for the first image in a Pictures-Linked movie, the context is registered, perhaps a relevant scene template or schema is activated fully (Gilboa and Marlatte, 2017), and then used to help link each image across the sequence. In contrast, the first image in a Pictures-Unlinked clip may be limited to just registering the context.

- The authors also state that "there was no difference apparent between the two conditions during the cue phase." Was this tested?

Yes, we examined the ERFs during the cue phase, as described in the Materials and Methods (p. 13) and Results sections (p. 16), and there was no difference in ERFs between the Pictures-Linked and Patterns-Linked conditions. As we now note in the revised manuscript, this shows that the ERF difference found at the first movie image did not merely bleed in from the cue period:

Discussion

(p. 18)

A notable feature of the results is that the only difference between scene and non-scene based events was during viewing of the first image frame, a point at which an event was yet to unfold. Participants were cued before each trial to inform them which condition was to come, but there was no difference apparent between the two conditions during the cue phase. Rather, the two event types diverged only when an event was initiated. This shows that the ERF difference found at the first movie image did not merely bleed in from the preceding cue period.

(p. 19)

In the Pictures-Linked condition, the participant knew from the preceding cue that the context depicted in the first image was going to endure for the entire clip, because the cue preceding each clip advised of the upcoming condition. Similarly, they knew that each image in the Pictures-Unlinked condition related to that image alone, and would not endure across the clip.

- One prominent difference between the picture-linked and the pattern-linked videos is that the former induce theory of mind processes, while the latter does not. How do the authors explain lack of increased activity for the picture-linked video in theory of mind and mentalizing regions?

Beyond the hippocampus, our results revealed greater involvement of a set of brain areas associated with Pictures-Linked more so than Patterns-Linked movies at the first image frame - the posterior parietal, inferior frontal, premotor, and cerebellar cortices. As we describe in the Discussion (pp. 21-22), these regions have been identified in numerous studies as part of a network that processes biological motion and the anticipation of intentional movement (Battelli et al., 2003; Rizzolatti and Craighero, 2004; Saygin et al., 2004; Fraiman et al., 2014). We suggested that Pictures-Linked movies were perceived as intentional, biological stimuli, while Patterns-Linked movies were not. Our Pictures-Linked stimuli share properties with simple point-light displays, in which a small number of moving lights (such as at the joints of a body) are sufficient for the viewer to interpret the movement as a particular behavior (such as dancing). While biological motion perception appears to relate to some aspects of theory of mind, they are not equivalent constructs (e.g. Rice et al., 2016; Meinhardt-Injac et al., 2018). For example, people with theory of mind deficits (e.g. in the context of autism) may demonstrate deficits in the perception of biological motion relative to controls but this may depend on whether emotional state information is required (Todorova et al., 2019). Whether there is a common neural circuitry underlying biological motion and theory of mind remains unclear. It is likely that the ability to perceive biological motion is required in order to make social judgements, but it is not the sole component of theory of mind processing (Fitzpatrick et al., 2018). We suggest that our simple, emotionally neutral event movies did not necessarily induce theory of mind processes and, consequently, engagement of brain areas associated with theory of mind was not increased for Pictures-Linked stimuli. We now allude to this point in the revised manuscript:

Discussion

(pp. 21-22)

Biological motion is often related to theory of mind. Could theory of mind explain the ERF differences between the Pictures-Linked and Patterns-Linked conditions? We feel this is unlikely given that brain areas typically engaged by theory of mind did not emerge in the analyses. Moreover, while biological motion perception appears to relate to some aspects of theory of mind, they are not equivalent constructs (e.g. Rice et al., 2016; Meinhardt-Injac et al., 2018). For example, people with theory of mind deficits (e.g. in the context of autism) may demonstrate deficits in the perception of biological motion relative to controls but this may depend on whether emotional state information is required (Todorova et al., 2019). Whether there is a common neural circuitry underlying biological motion and theory of mind remains unclear. It is likely that the ability to perceive biological motion is required in order to make social judgements, but it is not the sole component of theory of mind processing (Fitzpatrick et al., 2018). We suggest that our simple, emotionally neutral event movies did not necessarily induce theory of mind processes and, consequently, engagement of brain areas associated with theory of mind was not increased for Pictures-Linked stimuli.
